# Personal Liberty

**Published:** 1914-07

**Authors:** 


### Personal Liberty.

"Give me Liberty or give me death," said Patrick Henry, in a speech which was one of the determining factors in precipitating the American revolution. Edgar M. Cullen, retired Chief Justice of the Court of Appeals, in an address before the New York State Bar Association, calls attention to the encroachment by law on the liberties of the American people. "No trade or calling seems so limited . . . the advocates of no hobby or nostrum so few . . . as to be denied the privilege of having a new misdemeanor created." This sentence is our excuse for referring again to this matter in a medical journal. Probably no trade or calling has originated so many restrictive laws as has the medical profession. And it should be conceded that, to give liberty, in many instances, and especially in the matters in which it has been curtailed by laws originating in the medical profession or based on medical knowledge, means to give death, either to the one demanding the particular freedom of action, or to some one else.

On the other hand, it must be recognized that, in matters of personal liberty, the freedom of the individual in the United States is a sardonic joke. From Russia, west to China, there is no country in which the morals and health and actions of the individual are so personally conducted as in this reputed land of liberty. Hence, there is good reason to consider the matter seriously, before we are either so hedged in with restrictive legislation as to render conscience or self control or common sense unnecessary or have erected so complicated a structure of guiding rules that it will fall to pieces by its own weight and produce a state of anarchy.

Some of the matters considered by Judge Cullen are the following: Violation of the principles of the national and state constitutions in regard to the subordination of military to civil power, as in the West Virginia coal riots; the placing of Copperfield, Wash., under military law, by order of the governor's female secretary, because the civil authorities failed to close the saloons; the conviction of the Pioneer Press Co. for publishing an account of an execution, though admittedly in moderate and unsensational language; eugenic legislation, requiring medical certificate for marriage, regarding which, Judge Cullen points out the perfectly obvious fact that marriage is not a necessary prerequisite to propagation; the control of manufactures and sales of merchandise purely in the interests of competing industries; the recent attempt to inforce total abstinence.

It is a very delicate question to decide, when the interests of the public justify the restriction of the individual. The point of view changes also. The men who established this country considered the right to bear arms so important as to place it in the constitution. We have reached a point at which, on the whole, the man who does not go armed, is a good deal safer, not only to the community but to himself. But, so long as the item remains in the Constitution, it ought to be observed.

A vast amount of legislation deals with strictly moral matters, not involving direct and gross injury to others. We hesitate to say anything that would appear to uphold wrong doing but, here also, it is not always easy to distinguish between principle and prejudice. Solon, one of the most important law givers, conducted houses of prostitution for profit; Solomon another legal authority, would have been liable if modern laws had been in force, to so many convictions for bigamy that he would still be serving time for them, if alive. Virtus is clearly the original of our word virtue, but it meant such a different conception to the Romans that it is only rarely that it can be translated from the Latin by the same word. Theoretically, we concede to every individual the right to his own religion, or to no religion at all; but we have a mass of Sunday laws which prohibit all sorts of actions that are allowed on other days. Even those regulations which are based on the hygienic need of rest, including the restriction of night work and of hours of labor, often result in discharge of employees because the conditions are not practical. We no longer persecute the heretic, except in such indirect ways but we suffer the independent workingman to be ostracized, deprived of a means of livelihood and even assaulted and killed, for a closely comparable form of heresy. Even legally, the status of organized labor is beginning to be acknowledged as was formerly, that of the established church. Understand, we ualism, but it is a dangerous precedent to admit that a man has not the right to a minority opinion, even if it is foolish.

We should recognize that the contention that liberty is dearer than life is not merely one of oratory. It is actually better to have a slightly higher death rate with reasonable liberty, than to achieve the potential maximum of longevity, with the constant friction of restraint. We may go farther and claim that the potential maximum, as it appears at present, cannot be achieved if all the restrictions necessary to its accomplishment are inforced. The list of preventable deaths is decreasing, though not at so rapid a rate as might be desired. But, already, we find an increase in various death rates from degenerational diseases, an important factor in which, is increased strain and effort due to the friction of highly developed civilization. One of our friends and patients fell dead, from apoplexy, due to the sudden financial strain of meeting medical and hygienic requirements for the prolongation of his life. The increased tax necessary to prevent typhoid undoubtedly produces a contra-account, though fortunately of less amount, of deaths from increased stress of living. From the standpoint of pathology, we recognize clearly, the cumulative action of repeated, minor traumatisms. We are already far past the time when one of good intentions could go through life with the comfortable feeling that he need not bother about the law since the law would not trouble him. Just as an example, one of our professional friends has announced his intention of eliminating entirely from his armamentarium, several useful, almost indispensable drugs, simply on account of the red tape with which their use is surrounded. Another, a general practitioner too, has eliminated several infectious diseases for the same reason.

It is proper that entirely local problems should be dealt with according to local interests but, owing to the existence of national and state governments, a technical knowledge of law is required, not only in regard to technical legal and business matters but for ordinary conduct. This statement is true both as regards license to practice, regulation of traffic, hours of labor, etc. franchise, but with regard to purely moral matters on which no general geographic diffierence of opinion exists.

The principle that one man's opinion should not dictate another's conduct, ought to extend well toward the control of majority rule. Only an overwhelming majority should restrict the liberty of a negligible minority. Certainly, a small minority of fluent reformers should not be able, by influence of a legislative body, to control the majority. Again, the same principle should prevent legislative control of religious and moral issues, unless these are supported also by clearly defined issues of community welfare. Many feel that personal liberty involves the right to do what is unwise and wrong, even in their own estimation, providing that such a course does not directly endanger others. At any rate, it is exceedingly difficult to draw the line between legislative control of morals and personal welfare and the forcing of an arbitrary opinion of one person upon another. For example, as a total abstainer, the son and grandson of total abstainers, we are heartily in sympathy with the arguments in favor of total abstinence, but we question whether the forcible thrusting of these sentiments down the throats of a majority who are not total abstainers is justifiable, or whether it makes any difference whether the majority is on one side or the other.

